# Performance Optimization of Alkaline Multi-Industrial Waste-Based Cementitious Materials for Soil Solidification

**DOI:** 10.3390/ma17205077

**Published:** 2024-10-18

**Authors:** Xiaoli Wang, Xiancong Wang, Pingfeng Fu, Jinjin Shi, Miao Xu

**Affiliations:** 1 School of Civil and Resources Engineering, University of Science and Technology Beijing, Beijing 100083, China; xiaoliwang@ustb.edu.cn (X.W.); runner17@163.com (X.W.); 2 Cangzhou Municipal Engineering Company Ltd., Cangzhou 061000, China; czszjsk@126.com (J.S.); czszxm1989@163.com (M.X.); 3 Road Materials and Technology Engineering Research Center of Hebei Province, Cangzhou 061000, China

**Keywords:** cementitious material, industrial solid wastes, soil stabilization, central composite design, Rietveld method, hydration reaction

## Abstract

This study presents the development of eco-friendly cementitious materials for soil stabilization, based on alkaline multi-industrial waste (AMIW), using steel slag (SS), blast furnace slag (BFS), carbide slag (CS), fly ash (FA) and flue gas desulfurization gypsum (FGDG) as the raw materials. The optimal AMIW-based cementitious material composition determined through orthogonal experiments was SS:CS:FGDG:BFS:FA = 15:10:15:44:16. Central composite design (CCD) in response surface methodology (RSM) was employed to optimize the curing process parameters. The maximum 7-day unconfined compressive strength (7d UCS) was achieved under the optimal conditions of 18.51% moisture content, 11.46% curing agent content and 26.48 min of mix-grinding time. The 7d UCS of the AMIW-stabilized soil showed a 24% improvement over ordinary Portland cement (OPC)-stabilized soil. Rietveld refinement results demonstrated that the main hydration products of the stabilized soil were C-S-H and ettringite. After curing for 7 days to 28 days, the C-S-H content increased from 3.31% to 5.76%, while the ettringite content increased from 1.41% to 3.54%. Mercury intrusion porosimetry (MIP) and scanning electron microscopy (SEM) analysis revealed that with the extension of curing time, the pores of the stabilized soil become smaller and the structure becomes denser, resulting in an improvement in compressive strength.

## 1. Introduction

The acceleration of global urbanization and industrialization has led to an increase in the production of industrial solid waste, posing significant challenges for environmental protection, climate change and sustainable resource utilization [1,2,3]. Approximately 100 billion tons of natural resources are consumed globally each year, with 67 billion tons being converted into atmospheric pollutants such as carbon dioxide or turning into solid waste [4,5]. In 2019, China generated approximately 3.5 billion tons of industrial solid waste [6], accounting for 43% of the total output. However, on average, merely around 13% of solid waste is recycled globally [7], with landfilling and incineration as the primary disposal methods, exacerbating environmental pollution and resource waste [8,9,10,11]. Moreover, with the rapid advancement of road construction, the demand for base materials has surged, among which soil typically serves as the primary raw material in road base materials. However, the properties of soils vary significantly across different regions [12]. Due to insufficient compressive strength and water stability, many soils cannot be used for infrastructure construction without reinforcement, especially as road base materials [13,14]. Stabilization and solidification with cement and lime are a widely used technique for improving the mechanical properties of soil [15,16]. However, the production process of cement and lime causes significant environmental pollution issues. It is estimated that the production of cement accounts for about 8% of global CO_2_ emissions [17]. Thus, the development of eco-friendly, low-carbon materials for soil solidification is urgently needed.

Supplementary cementitious materials generally consist of industrial by-products, natural pozzolans and reactive minerals that exhibit hydraulic or pozzolanic characteristics [18,19,20]. They can effectively overcome the shortcomings of conventional soil curing agents such as cement and lime, including high energy consumption, high pollution, and poor durability, and are considered to be an ideal substitute for traditional cement [21,22,23,24,25]. Recently, utilizing alkaline industrial solid waste as cementitious materials for soil stabilization has become an effective way to solve resource waste and environmental pollution, while also enhancing the effective utilization of industrial solid waste [6,26,27,28]. Alkaline industrial wastes are characterized by their containing mainly silicates, aluminates, calcium or magnesium oxides and aluminosilicates which dissolve or hydrate and create an alkaline environment [29,30]. Typical alkaline industrial solid wastes include carbide slag (CS), fly ash (FA), blast furnace slag (BFS), steel slag (SS) and flue gas desulfurization gypsum (FGDG) [31]. CS is the residual slag generated by the hydrolysis of calcium carbide during the production of acetylene [32]. The main component of CS is calcium hydroxide, which is highly alkaline (pH > 13) and can be used as a substitute for lime [33]. It can be used as alkaline activators because of their ability to provide Ca^2+^ and OH^−^ [34]. FA is the main solid waste generated from thermal power plants [35]. BFS and SS are the solid wastes generated in ironmaking and steelmaking processes [36,37]. SS has a similar composition to cement and can be used as a substitute for cement [38]. FGDG is a secondary product formed during the flue gas desulfurization process in thermal power plants [39]. The primary chemical components of these industrial solid wastes are SiO_2_, Al_2_O_3_, CaO and MgO, demonstrating high pozzolanic activity and showing great potential in soil solidification. Several studies have reported the utilization of cementitious materials based on industrial solid waste for soil stabilizer [22,27,40,41]. The conventional X-ray powder diffraction (XRD) analysis is commonly used for phase analysis of hydration products. However, due to the complex phase and crystal structure, strong overlapping diffraction peaks hinder quantitative analysis. The Rietveld refinement method is an effective tool for qualitative and quantitative phase analysis [42]. Few studies have concentrated on the investigation of hydration products with the application of the Rietveld method. Moreover, the influence of certain curing parameters on the effectiveness of solidification can often be underestimated, such as the following: moisture content, dosage of the curing agent, and mix-grinding time.

This study presents a proposal for the development of eco-friendly alkaline multi-industrial waste (AMIW)-based cementitious materials for soil stabilization by using multiple industrial solid wastes: steel slag (SS), blast furnace slag (BFS), carbide slag (CS), fly ash (FA) and flue gas desulfurization gypsum (FGDG) as the main raw materials. Optimization of material formulation was obtained through orthogonal experimental design. The central composite design (CCD) method in response surface methodology (RSM) [43] was used to evaluate the effects of moisture content, the dosage of the curing agent and mix-grinding time on the curing effect of AMIW-based soil stabilizer, and obtain optimal curing parameters. The important mechanical properties of stabilized soil are analyzed, such as unconfined compressive strength. The Rietveld method is employed to analyze the phase changes and quantify the hydration products, as well as to investigate the hydration mechanism. The pore structure and microstructure of the stabilized soil are investigated and the synergistic mechanism of hydration reaction is discussed.

## 2. Materials and Methods

### 2.1. Materials

**Figure 1:**
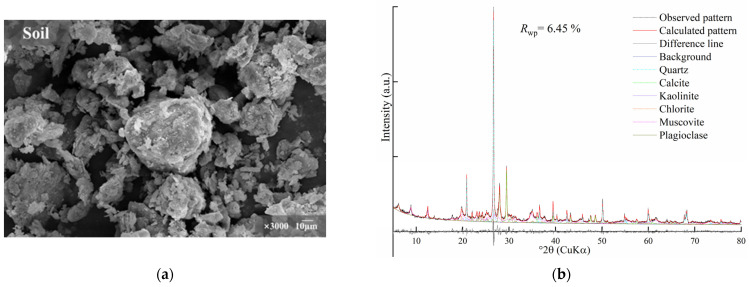
(**a**) SEM image and (**b**) Rietveld refinement of test soil.

The soil used in this research was sourced from cultivated land in Cangzhou, China, at a depth of approximately 1 to 1.5 m. The fundamental physical properties of the test soil were determined according to the Chinese standard for the Soil Test Method (GB/T50123-2019) [44]. From the results (Table 1), it was apparent that the test soil had a low plasticity index and poor stability when in contact with water. An SEM image and the XRD pattern of the test soil are presented in Figure 1. Qualitative and quantitative analysis of phases in the test soil were determined by the Rietveld method. According to the results (Table 2), the R_wp_ value was 7.71% and the measured patterns and calculated patterns demonstrated a high degree of correspondence. The main phases of test soil were quartz (25.76%), chlorite (23.03%), plagioclase (14.23%), muscovite (13.77%) and calcite (9.95%). The SEM image indicates that soil particles were irregularly shaped, unevenly distributed and had an aggregate or flake structure.

**Figure 2:**
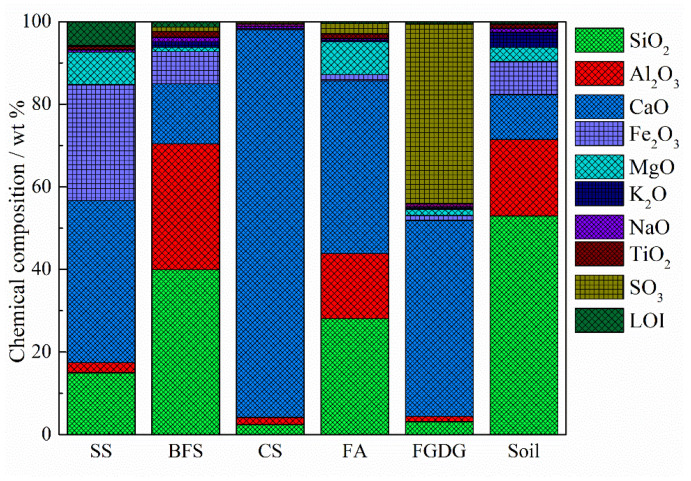
Chemical composition of test soil and raw materials.

**Figure 3:**
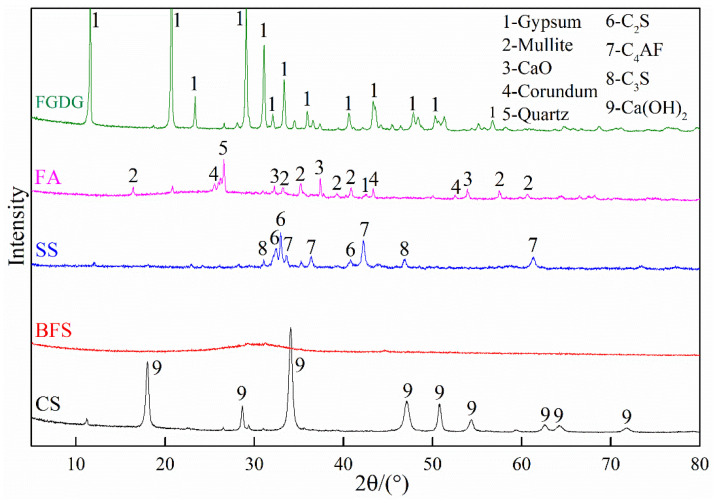
XRD patterns of FGDG, FA, SS, BFS and CS.

SS and BFS were collected from China Railway Co., Ltd. (Cangzhou, China), with a specific surface area of 511 m^2^/kg and 545 m^2^/kg, respectively; FA and FGDG were obtained from the Huanghua Power Plant (Cangzhou, China), with a specific surface area of 548 m^2^/kg and 540 m^2^/kg, respectively; CS was supplied by Jinniu Chemical Co., Ltd. (Cangzhou, China) in Hebei Province, China, with a specific surface area of 578 m^2^/kg. The chemical and phase compositions of the solid wastes were detected via XRF and XRD analysis. The results are displayed in Figure 2 and Figure 3. The dominant chemical components of these industrial solid wastes were SiO_2_, CaO and Al_2_O_3_. In addition, SS contained 28.01% FeO and FGDG contained 43.34% SO_3_. The major phases of FA consisted of mullite, quartz, CaO and corundum; SS contained mainly C_2_S, C_3_S and C_4_AF; FGDG and CS contained gypsum and CaOH, respectively; BFS had an amorphous structure.

### 2.2. Methods

#### 2.2.1. Specimen Preparation

The test soil and raw materials were thoroughly dried before the experiment. The AMIW-based cementitious material for soil stabilization was prepared by mixing CS, SS, FGDG, BFS and FA in specific proportions. The optimal proportion was obtained by orthogonal experiments. The compressive strength of pure paste and blended paste specimens with stabilizer/soil were investigated. The optimal curing agent content, moisture content and mix-grinding time were determined by the CCD method. After these raw materials were thoroughly mixed, we added them to the test soil and mixed them again. Then, an amount of water was added to the mixture and stirred quickly until uniform, and we placed the mixture into sealed airtight bags to be stored for 24 h to prevent moisture loss. According to Chinese standard JTGE51-2009 (Test Methods of Materials Stabilized with Inorganic Binders for Highway Engineering) [45], the specimens were 50 mm × 50 mm. The specimens were then stored in an environment with a relative humidity exceeding 95% and a temperature of 20 ± 2 °C until the specified curing time was completed. Once the curing process was completed, the samples were analyzed for compressive strength and microstructure.

#### 2.2.2. Orthogonal Experimental Design

An orthogonal experimental design was used to optimize the proportions of raw materials. The mass ratio of CS, SS, FGDG and BFS were set as four factors and the levels are shown in Table 3. The mass proportion of FA can be calculated by the following formula: FA% = 100% − (CS% + SS% + FGDG% + BFS%). Based on the orthogonal experimental design table of L16 (4^4^), a total of 16 subjects were evaluated. The details are listed in Table 4.

Range analysis was applied to assess the results of the orthogonal experiments and determine the factors’ sensitivity to the unconfined compressive strength. The influence degree R and the average value ki are calculated as follows:

(1) $$k_{i,j}=\bar{P_{0.95}(k_{i,j})}\;\;(i=factors;j=levels)$$

(2) $$R=\max\left\{k_{i,1},k_{i,2},\;k_{i,3}\text{⋯}\right\}-\min\left\{k_{i,1},k_{i,2},\;k_{i,3}\text{⋯}\right\}$$

where k_i,j_ is the average value of test results at level j of factor i; the impact level R indicates the effect of each factor on the index. The larger the R value, the more significant the influence of this factor on the experiment results.

#### 2.2.3. Central Composite Design (CCD)

**Figure 4:**
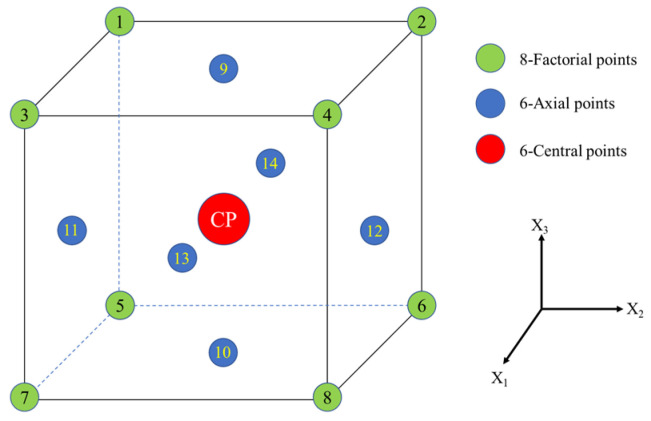
Point positions of in 2^3^ factorial design.

Central composite design (CCD) is a commonly used approach in Response Surface Methodology (RSM) [46]. The mathematical models frequently employed in RSM include linear, quadratic and response surface models. Analysis of these models enables the identification of the best combination of levels for factors. In general, CCD encompasses 2n axial test points (where n signifies the count of experimental factors) and additional 2^n^ factorial test points. Furthermore, the empirical error is assessed through the utilization of repeated central test points. The axial test point serves the purpose of screening analysis and assessing model prediction variance, while the centrally repeated test point significantly contributes to the autonomous assessment of empirical error. The 2^n^ factorial test points are designated as +1 and −1, while the 2n axial test points are characterized by coding such as (±α, 0, …, 0), (0, ±α, 0, …, 0), and so forth (Table 5). The codes for repeated central test points are represented as (0, 0, …, 0). The distance from the center at which to position the axial runs within the coded scale is referred to as the alpha values [47,48]. In this study, the influence of three independent variables, including curing agent content, mix-grinding time, and moisture content, on the unconfined compressive strength were studied by the CCD method using the Design-Expert 13 program. A 2^3^ CCD model was generated (Figure 4). The examination of the three experimental factors requires 8 factorial test points and 6 axial test points. Three levels for each factor are illustrated in Table 6. Additionally, 6 central test points are designated for error estimation. As depicted in Figure 3, a total of 20 tests were conducted (Table 7).

#### 2.2.4. Test Methods

Unconfined compressive strength (UCS) was measured using an electro-hydraulic universal testing machine (WAW-1000B, Jinan, China) in accordance with the GB/T17671 China standard [49]. The phase composition of samples was determined by X-ray powder diffraction (XRD) method with a D8 ADVANCE X-ray diffractometer (Bruker, Ettlingen, Germany), employing Cu Kα radiation (λ = 0.1542 nm) at 40 kV and 40 mA. The scanning angle varied from 5° to 80°, with a step size of 0.04°. The Rietveld refinement method was employed in this study for the quantification of mineral phases of samples. The BGMN software (version 5.1.8) was used for Rietveld refinement analysis. The microstructure of samples was examined using scanning electron microscopy (SEM, JSM-6701F, Tokyo, Japan). The pore structure of samples was analyzed by Autopore IV 9510 Mercury intrusion porosimetry (MIP, Shanghai, China).

## 3. Results and Discussion

### 3.1. Orthogonal Test Results Analysis

Table 8 lists the results of the orthogonal experiment and range analysis. The maximum 7-day unconfined compressive strength (7d UCS) is 1.72 MPa. The value of the range is utilized to determine the influence level of various factors on the 7d UCS. According to the result of range analysis, the degree of influence of each factor on 7d UCS is SS > CS > BFS > FGDG. The content of SS has the greatest impact on 7d UCS, and BFS has the least impact. The optimal proportion of raw materials is SS_1_CS_4_FGDG_3_BFS_3_, and the corresponding percentage content is 15% SS, 10% CS, 15% FGDG and 44% BFS. The content of FA was calculated to be 16%.

**Figure 5:**
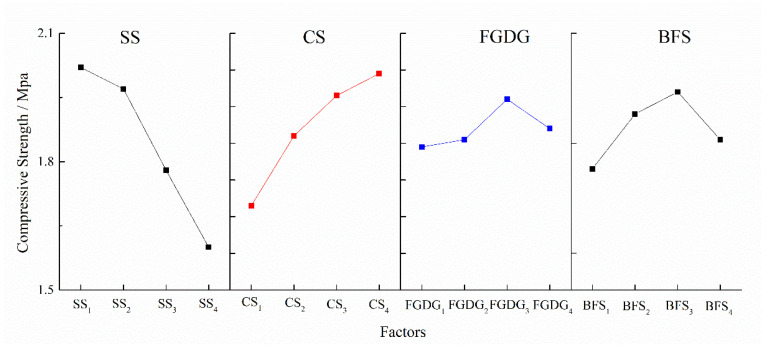
Effect curve of factors on compressive strength.

Figure 5 shows the effect curve of SS, CS, FGDG and BFS on compressive strength. The results indicate that as the SS content increases, the 7d UCS decreases, suggesting that SS has a negative impact on compressive strength. In contrast, as the CS content increases, the 7d UCS rises, indicating that CS has a positive effect on compressive strength. However, when the contents of FGDG and BFS increase, the 7d UCS first increases and then decreases.

### 3.2. Optimization of Curing Conditions

#### 3.2.1. Fitting Model and Statistical Analysis

The 7d UCS of stabilized soil and the correlation between three independent variables, including curing agent content, mix-grinding time and moisture content, were investigated by the CCD method using the Design-Expert 13 program. The predictive and experimental values of 7d UCS are shown in Table 7. Fitting models for the three independent variables against the 7d UCS response were developed. The evaluation of the four fitted models is shown in Table 9. Models are considered appropriate for data fitting when they demonstrate a lower sequential *p*-value and lack of fit *p*-value, and a higher adjusted R^2^ value and predicted R^2^ value. As seen from Table 9, the quadratic model with sequential *p*-value < 0.0001 (*p*-value > 0.05 insignificant, *p*-value < 0.05 significant, *p*-value < 0.01 extremely significant) and an adjusted R^2^ value of 0.9398 was considered to have a notably strong degree of fit and a high significance. Therefore, a multiple quadratic regression equation was employed to investigate the interrelationship between the response (7d UCS) and the variable factors.

The quadratic regression model depicting the relationship between the three independent variables and the 7d UCS response was derived through regression analysis (Equation (3)). Analysis of variance (ANOVA) was used to assess the quality of the fitted models. Table 10 presents the ANOVA for the 7d UCS response surface quadratic model. Typically, the smaller the *p*-value and the larger the F-value, the higher the significance of the respective coefficients. As shown in Table 10, the *p*-value and F-value of the model are <0.0001 and 33.95, respectively, indicating that the model is statistically significant. The ANOVA results suggest that the ranking of the influence of the three factors on the 7d UCS of stabilized soil is as follows: curing agent content (x_2_) > moisture content (x_1_) > mix-grinding time (x_3_). The analysis result of the reliability test for the quadratic model is shown in Table 11. The values of R^2^ reflect the degree of model fitting. A higher R^2^ value indicates a stronger correlation between the predicted and actual values. According to the results, the values of R^2^ and adjusted R^2^ were 0.9683 and 0.9398, respectively, and the gap between the adjusted R^2^ and predicted R^2^ was less than 0.2, indicating that the regression equation could characterize the relationship between the factors and the 7d UCS effectively, exhibiting a high level of reliability. The coefficient of variation (C.V.) was 3.27 (<5%), demonstrating the high accuracy of the selected model.

(3) $$Y_{7d}=1.65+0.0412x_{1}+0.1857x_{2}+0.0375x_{3}+0.021x_{1}x_{2}\;-\;0.0218x_{1}x_{3}\;-\;0.0016x_{2}x_{3}\;-\;0.0586x_{1}^{2}-\;0.1123x_{2}^{2}\;-\;0.0403x_{3}^{2}$$

#### 3.2.2. Optimization of Curing Process Parameters

**Figure 6:**
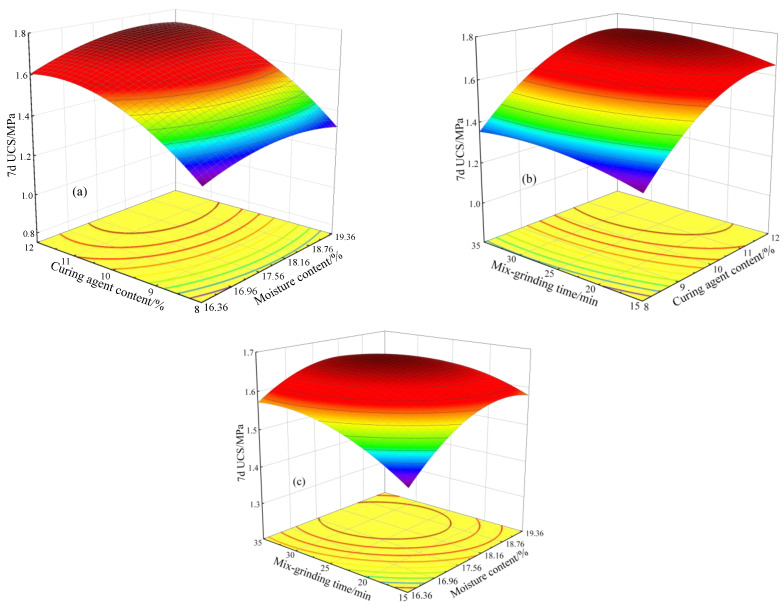
Response surfaces and contour plot for experimental models. (**a**) the effect of moisture content and curing agent content on 7d UCS; (**b**) the effect of curing agent content and mix-grinding time on 7d UCS; (**c**) the effect of moisture content and mix-grinding time on 7d UCS.

Figure 6 presents the response surface and contour map of the impact of two interacting factors on 7d UCS of stabilized soil. As observed in Figure 6a,c, with the dosage of moisture content and mix-grinding time held constant at a medium level (17.86% and 25 min), the 7d UCS exhibited an increasing trend as the curing agent content increased. Figure 6a shows that at lower moisture levels, increasing the dosage of the curing agent could enhance the 7d UCS, though it did not reach the peak value. Conversely, increasing the moisture content while maintaining a low curing agent dosage did not notably improve the 7d UCS of the stabilized soil. Figure 6c exhibits a similar trend: when the mix-grinding time took a lower value, by increasing the amount of curing agent, the 7d UCS increased, but it did not reach the maximum value; while maintaining a low curing agent dosage, increasing the mix-grinding time did not significantly improve the 7d UCS. Hence, this demonstrates that within the interaction between moisture content and curing agent content, mix-grinding time and curing agent content, the impact of curing agent content on 7d UCS is more obvious. Figure 6b demonstrates that when the moisture content is set to the median value (17.86%), the 7d UCS was gradually improved with the extension of the mix-grinding time; when the mix-grinding time is set to the median value (25 min), the 7d UCS was gradually increased with the extension of the moisture content. It is suggested that appropriately increasing the moisture content in the soil and extending the mix-grinding time can improve the compressive strength.

The optimal curing process parameters were obtained using the numerical optimization algorithm in the Design-Expert 13 software. Based on the prediction results of the quadratic regression model, the maximum 7d UCS of stabilized soil (1.75 MPa) was obtained under the optimal conditions of 18.51% moisture content, 11.46% curing agent content, and 26.48 min of mix-grinding time. The test result of the verification experiments is listed in Table 12. The measured 7d UCS value closely matched the predicted value under optimal curing conditions. The absolute relative deviation is 1.15% (<5%), indicating the model has a high accuracy.

### 3.3. Compressive Strength Test

**Figure 7:**
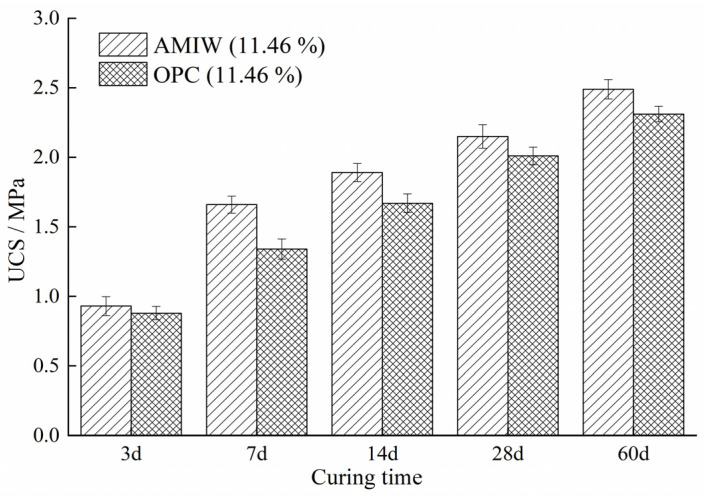
UCS of curing agent- and cement-stabilized soil.

To evaluate the mechanical performance differences between AMIW-stabilized soil and ordinary Portland cement (OPC)-stabilized soil, the UCS of the two stabilized soils was evaluated under the same optimal conditions (11.46% curing agent content, 18.51% moisture content and 26.48 min mix-grinding time). Figure 7 shows the UCS of stabilized soil at various curing ages. The compressive strength of stabilized soil using AMIW and OPC at a 3-day curing age was similar (0.93 MPa and 0.88 MPa); the UCS of AMIW-stabilized soil was slightly greater. The 7d UCS of AMIW-stabilized soil was 1.66 MPa, which is 24% higher than that of OPC stabilized soil (1.34 MPa), meeting the requirements of Technical Guidelines for Highway Roadbases Construction (JTG/TF20-2019) [50]. Subsequently, with the extension of curing time, the UCS showed an increasing trend, and the UCS of AMIW group was greater than that of OPC group. Following the 60-day curing period, the UCS of the soils stabilized with AMIW and OPC were 2.49 MPa and 2.31 MPa, respectively.

## 4. Microscopic Characteristics of the Hydration Products

### 4.1. Rietveld Refinement Analysis

**Figure 8:**
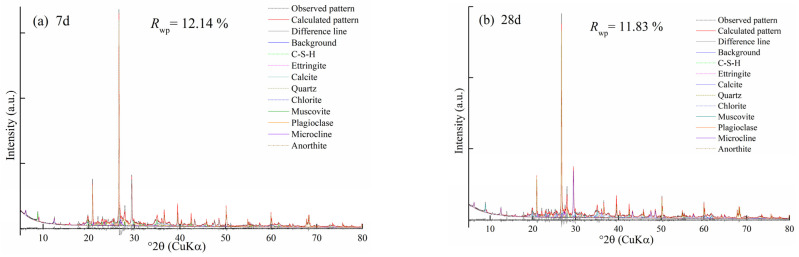
Rietveld refinements of AMIW-stabilized soil at curing age of (**a**) 7 days and (**b**) 28 days.

The Rietveld refinement method was used to determine the mineral composition and content of hydration products. The quality of refinement was measured by the weighted profile *R* factor (*R*_wp_). The refinement of AMIW-stabilized soil at 7 and 28 days of curing showed a good agreement between the measured and calculated patterns (Figure 8); the *R*_wp_ values were 11.83% and 12.14%, respectively. The refinement results showed that quartz (SiO_2_), chlorite ((Fe, Mg)_5_Al(Si_3_Al)O_10_(OH)_8_), muscovite (KAl_2_(AlSi_3_O_10_)(OH)_2_), plagioclase ((Na,Ca)(Si,Al)_4_O_8_) and calcite (CaCO_3_) were found to be the primary phases in the stabilized soil. The main hydration products were ettringite (3CaO·Al_2_O_3_·3CaSO_4_·32H_2_O) and calcium silicate hydrate (C-S-H, CaO·SiO_2_·nH_2_O), both of which played an active role in the development of compressive strength [31,36]. The refinement results are presented in Table 13. It is noteworthy that the amounts of ettringite and C-S-H increased gradually with the increasing curing periods. Following 7 and 28 days of curing, the C-S-H content rose from 3.31% to 5.76%, and the content of ettringite increased from 1.41% to 3.54%, representing increases of 74.02% and 151.06%, respectively. This indicates that as the hydration reaction proceeds, the content of hydration products increases continuously, resulting in a denser internal configuration of the stabilized soil, thereby increasing the compressive strength, which corresponds to the result of UCS. The content of quartz and calcite remained nearly constant after curing for 7 and 28 days, indicating that they did not participate in the hydration reaction. Furthermore, no diffraction peaks of gypsum, C_2_S and C_3_S were found, suggesting that they were involved in the hydration reaction at an early stage and had been consumed.

### 4.2. Pore Structure Analysis

The strength of AMIW-stabilized soil is closely related to its pore structure [51]. The MIP test method was used to analyze the porosity, bulk density, apparent density and pore size distribution data of the stabilized soil at different curing ages. The results are shown in Table 12. The results show that as the stabilized soil ages, the total pore volume and porosity decrease. However, the compressive strength increases with the prolongation of curing time, which illustrates the negative correlation between the strength of cured soil and these factors. On the contrary, due to the higher density of hydration products, both bulk density and apparent density increase with curing time, leading to an increase in compressive strength. Additionally, the average pore diameter tends to become smaller as the curing time increases. The average pore diameters after curing for 7 days, 28 days and 60 days were 73.36 nm, 42.16 nm and 37.36 nm, respectively.

**Figure 9:**
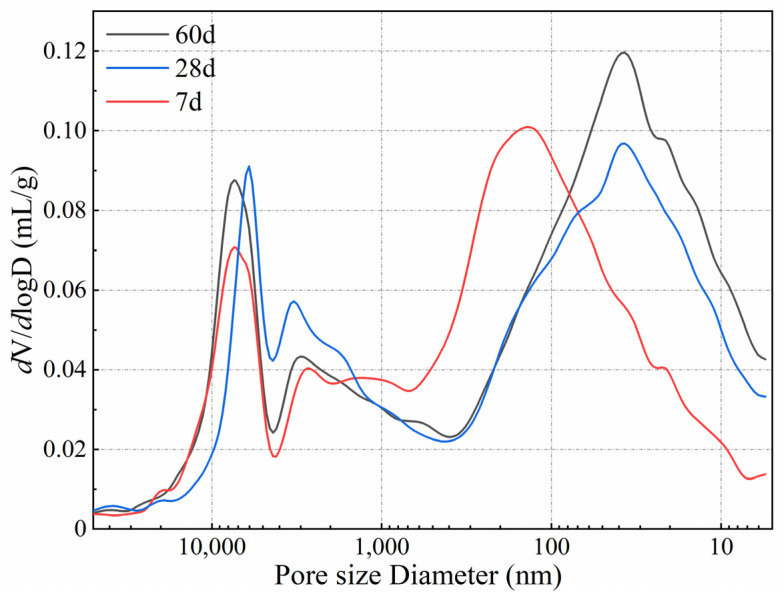
Pore size and log-differential volume curve plots of AMIW-stabilized soil.

Figure 9 presents the cumulative porosity and log-differential volume curves for the stabilized soil after 7, 28, and 60 days of curing. In the logarithmic differential and cumulative porosity plots, the pore size shifts from large to small as the curing time increases. The pore size corresponding to the highest peak represents the most widely distributed pore size range in the stabilized soil. It is commonly believed that the smaller the critical pore diameter, the finer the pore structure [45]. From Figure 8, it is clear that as the curing time increases, the pores of the consolidated soil become smaller and the structure becomes denser. The results of SEM also confirmed this conclusion. It is worth noting that the number of pores increased significantly after 60 curing days compared with 28 curing days, indicating an increase in density. This is consistent with the conclusion in Table 14. The reason may be that the hydration reaction continues and the hydration products gradually increase, leading to a denser internal structure of the stabilized soil system.

### 4.3. SEM Analysis

**Figure 10:**
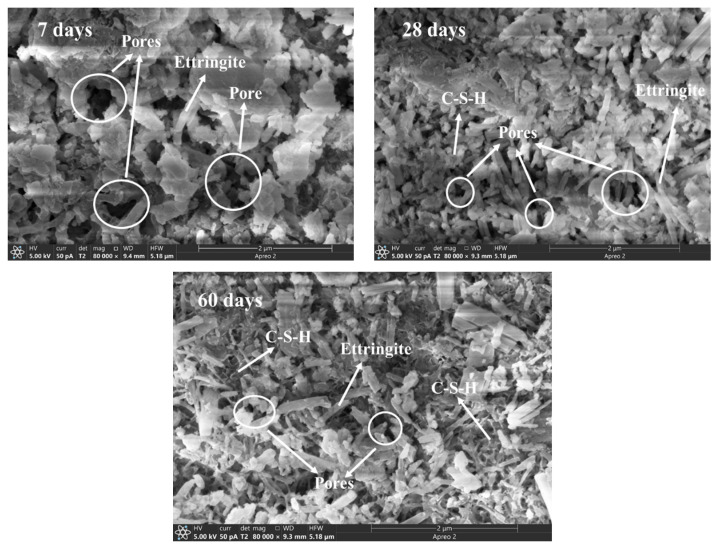
Microstructure of AMIW-stabilized soil at different curing times.

The microstructure of AMIW-stabilized soil curing for 7, 28 and 60 days is displayed in Figure 10. It can be seen that after 7 days of curing, large-sized pores can be observed, and the structure of the stabilized soil was loose at this time. After 28 days of curing, the number of pores decreased significantly, and their size became smaller, and the structure was relatively dense. Following 60 days of curing, the trend of diminishing large pores continued and these pores turned into many small pores, resulting in a denser structure of the stabilized soil. The changes in the microstructure of the stabilized soil are consistent with the conclusions of porosity and pore diameter of the MIP test results (Table 12). During the initial curing period of the first 7 days, the stabilized soil contains a relatively small amount of ettringite due to the short hydration reaction time. Nevertheless, as the curing time extended and the hydration reaction continued, the amount of ettringite and C-S-H increased substantially. After curing for 28 days, the generated ettringite and C-S-H became intertwined with one another, filling the pores. This structure is beneficial to the improvement of UCS. After 60 days of hydration, the stable three-dimensional network structure formed by ettringite and C-S-H gel exhibited higher density and efficiently occupied the pores within the stabilized soil structure. This process strengthens the compactness of soil structure and provides an explanation for the increase in compressive strength as the curing time extends (Figure 7).

### 4.4. Synergistic Mechanism of Hydration Reaction

**Figure 11:**
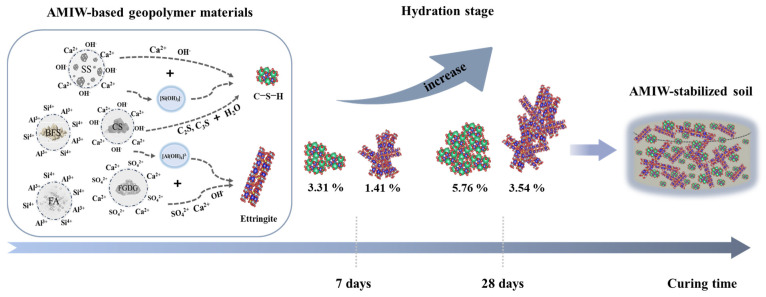
Schematic diagram of hydration reaction synergistic mechanism.

From the characterization of AMIW-stabilized soil, it is evident that the improvement of UCS under the synergistic action of SS, BFS, CS, FA and FGDG in the raw materials is mainly attributed to the increase in the amount of hydration products and changes in the microstructure. The primary component of CS is Ca(OH)_2_ (Figure 2), which generates Ca^2+^ and OH^−^ after hydrolysis, providing an alkaline environment for the system (Equation (4)). C_2_S and C_3_S in SS are hydrolyzed in the early stage of the hydration reaction to generate C-S-H and Ca(OH)_2_, providing more Ca^2+^ and OH^−^ to the system (Equations (5) and (6)). Subsequently, Si-O and Al-O bonds of SiO_2_ and Al_2_O_3_, the main components of BFS and SS, were broken under alkaline conditions, resulting in the liberation of SiO_3_^2−^ and AlO^2−^ (Equations (7) and (8)). These ions further recombine with OH^−^ to form [H_3_SiO_4_]^−^ and [Al(OH)_6_]^3−^ (Equations (9) and (10)). [H_3_SiO_4_]^−^ reacted with Ca^2+^ released from FA and CS to generate C-S-H; [H_3_AlO_4_]^2−^ reacted with Ca^2+^ and SO_4_^2−^ in FGDG to form ettringite (Equations (11) and (12)). Figure 11 presents the hydration mechanism and quantitative change in the primary hydration products.

(4) $${Ca\left(OH\right)}_{2}\to {2Ca}^{2+}+{OH}^{-}$$

(5) $$C_{2}S+H_{2}O\to m\mathrm{CaO}\cdot SiO_{2}\cdot nH_{2}O+{Ca\left(OH\right)}_{2}$$

(6) $$C_{3}S+H_{2}O\to m\mathrm{CaO}\cdot SiO_{2}\cdot nH_{2}O+{Ca\left(OH\right)}_{2}$$

(7) $${Al}_{2}O_{3}+{OH}^{-}\to {{AlO}_{2}}^{-}$$

(8) $$SiO_{2}+{OH}^{-}\to {{SiO}_{3}}^{2-}$$

(9) $${{AlO}_{2}}^{-}+H_{2}O+{OH}^{-}\to {\left[{\mathrm{Al}(\mathrm{OH})}_{6}\right]}^{3-}$$

(10) $${{SiO}_{3}}^{2-}+H_{2}O+{OH}^{-}\to {\left[H_{3}{SiO}_{4}\right]}^{-}$$

(11) $$2{\left[{\mathrm{Al}(\mathrm{OH})}_{6}\right]}^{3-}+{{6Ca}^{2+}+{3SO}_{4}}^{2-}+26H_{2}O\to \;\mathrm{CaO}\cdot {\mathrm{Al}}_{2}O_{3}\cdot 3\mathrm{CaS}O_{4}\cdot 32H_{2}O$$

(12) $${\left[H_{3}{SiO}_{4}\right]}^{-}+{mCa}^{2+}+H_{2}O\to m\mathrm{CaO}\cdot SiO_{2}\cdot nH_{2}O$$

## 5. Conclusions

In this study, an environmentally friendly AMIW-based cementitious material for soil stabilization was prepared using alkaline industrial solid wastes such as SS, BFS, CS, FA and FGDG. The CCD method was used to evaluate the impact of moisture content, the dosage of the curing agent and mix-grinding time on the UCS of the stabilized soil. The hydration characteristics of stabilized soil were determined by the Rietveld refinement method and SEM and MIP test methods. The main conclusions are summarized as follows:

(1) The optimal mass ratio for preparing AMIW-based cementitious material compositions is SS:CS:FGDG:BFS:FA = 15:10:15:44:16. The UCS of the stabilized soil improved as the curing time progressed. CS positively influences compressive strength, whereas SS negatively affects compressive strength.
(2) A quadratic model was developed to predict the effect of three curing process parameters on 7d UCS. The analysis results of ANOVA and the reliability test for the quadratic model indicate that the model is statistically significant and has high reliability and accuracy. The optimal conditions for curing were as follows: moisture content of 18.51%, curing agent content of 11.46% and mix-grinding time of 26.48 min. Under these conditions, the 7-day UCS of AMIW-stabilized soil was 24% greater than that of OPC-stabilized soil. Following 60 days of curing, the UCS of AMIW-stabilized soil could reach 2.79.
(3) The Rietveld refinement results indicated that the main hydration products were C-S-H and ettringite. As the curing period extended from 7 days to 28 days, the C-S-H content rose from 3.31% to 5.76% and the ettringite content increased from 1.41% to 3.54%. MIP and SEM analysis showed that with the extension of curing time, the pores of the stabilized soil became smaller, ettringite and C-S-H grew intertwined and filled the pores, and the structure became denser, promoting the enhancement of the UCS.

## Figures and Tables

**Table 1:** Characteristics of test soil.

Initial Water Content/(%)	Optimum Water Content/(%)	Maximum Dry Density/(g·cm^−3^)	Liquid Limit /(%)	Plastic Limit /(%)	Plasticity Index
9.42	16.09	1.86	40.31	24.92	15.30

**Table 2:** Rietveld refinement result of test soil.

	Quartz	Muscovite	Calcite	Chlorite	Plagioclase	Microinte	Anorthite	R_wp_/(%)	R_exp_/(%)
Phases contents/(wt.%)	25.76 (4) ^a^	13.77 (3)	9.95 (2)	23.03 (6)	14.23 (4)	6.56 (3)	6.70 (5)	7.71	4.29

^a^ Standard deviation.

**Table 3:** Factors and levels of orthogonal test.

Levels	Factors			
	Mass Proportions/(%)			
	CS	SS	FGDG	BFS
1	4	15	9	36
2	6	20	12	40
3	8	25	15	44
4	10	30	18	48

**Table 4:** Schemes of orthogonal experimental design.

No.	Levels				Mass Proportion of FA/(%)
	SS	CS	FGDG	BFS	
1	1	1	1	1	36
2	1	2	2	2	27
3	1	3	3	3	18
4	1	4	4	4	9
5	2	1	2	3	23
6	2	2	1	4	18
7	2	3	4	1	17
8	2	4	3	2	12
9	3	1	3	4	13
10	3	2	4	3	10
11	3	3	1	2	15
12	3	4	2	1	12
13	4	1	4	2	12
14	4	2	3	1	13
15	4	3	2	4	2
16	4	4	1	3	3

**Table 5:** Correlation between coded value and actual value of factors.

Code	The Actual Level of Factors
−α	X_min_
−1	[(μ + 1) · X_min_ + (μ − 1) · X_max_]/2μ
0	(X_min_ + X_max_)/2
+1	[(μ − 1) · X_min_ + (μ + 1) · X_max_]/2μ
+α	X_max_

Note: μ is 2^n/4^, n is the number of factors, and n = 3.

**Table 6:** Independent variables and their levels for CCD.

Factors	Level		
	−1	0	1
Moisture content/(%)	16.36	17.86	19.36
Curing agent content/(%)	8	10	12
Mix-grinding time/(min)	15	25	35

**Table 7:** Design matrix and results of CCD.

Sample	Moisture Content	Curing Agent Content	Mix-Grinding Time	7d UCS/MPa	
				Predictive	Actual
1	−1.000	−1.000	−1.000	1.17	1.16
2	1.000	−1.000	−1.000	1.26	1.30
3	−1.000	1.000	−1.000	1.51	1.46
4	1.000	1.000	−1.000	1.68	1.68
5	−1.000	−1.000	1.000	1.30	1.29
6	1.000	−1.000	1.000	1.29	1.34
7	−1.000	1.000	1.000	1.62	1.58
8	1.000	1.000	1.000	1.69	1.71
9	−1.682	0.000	0.000	1.42	1.48
10	1.682	0.000	0.000	1.56	1.49
11	0.000	−1.682	0.000	1.02	0.99
12	0.000	1.682	0.000	1.65	1.68
13	0.000	0.000	−1.682	1.48	1.48
14	0.000	0.000	1.682	1.60	1.59
15	0.000	0.000	0.000	1.65	1.66
16	0.000	0.000	0.000	1.65	1.67
17	0.000	0.000	0.000	1.65	1.61
18	0.000	0.000	0.000	1.65	1.70
19	0.000	0.000	0.000	1.65	1.63
20	0.000	0.000	0.000	1.65	1.66

**Table 8:** Result of orthogonal experiment and range analysis.

No.	7d UCS/MPa			
1	1.23			
2	1.48			
3	1.72			
4	1.6			
5	1.38			
6	1.41			
7	1.44			
8	1.68			
9	1.2			
10	1.39			
11	1.4			
12	1.36			
13	1.08			
14	1.17			
15	1.22			
16	1.33			
Levels	SS	CS	FGDG	BFS
*k*1	2.02	1.63	1.79	1.73
*k*2	1.97	1.82	1.81	1.88
*k*3	1.78	1.93	1.92	1.94
*k*4	1.6	1.99	1.84	1.81
Range	0.42	0.36	0.09	0.21
Ranking	SS > CS > BFS > FGDG			
Optimum theme	SS_1_CS_4_FGDG_3_BFS_3_			

**Table 9:** Comprehensive analysis of 7d UCS by various models.

Source	Sequential *p*-Value	Lack of Fit *p*-Value	Adjusted R^2^	Predicted R^2^	Evaluate
Linear	0.0004	0.0013	0.6057	0.5128	-
2FI (2-factor interaction)	0.9414	0.0007	0.5287	0.1866	-
Quadratic	<0.0001	0.0739	0.9398	0.7975	Suggested
Cubic	0.0258	0.9755	0.9803	0.9908	Aliased

**Table 10:** ANOVA table for 7d UCS response surface quadratic model.

Source	Sum of Squares	d*f*	Mean Square	*F*-Value	*p*-Value	
Model	0.7442	9	0.0827	33.95	<0.0001	significant
*x*_1_—moisture content	0.0232	1	0.0232	9.54	0.0115	
*x*_2_—curing agent content	0.4709	1	0.4709	193.31	<0.0001	
*x*_3_—mix-grinding time	0.0192	1	0.0192	7.9	0.0184	
*x*_1_ *x*_2_	0.0035	1	0.0035	1.45	0.2563	
*x*_1_ *x*_3_	0.0038	1	0.0038	1.56	0.2398	
*x*_2_ *x*_3_	0	1	0	0.0084	0.9288	
*x*_1_^2^	0.0494	1	0.0494	20.29	0.0011	
*x*_2_^2^	0.1817	1	0.1817	74.59	<0.0001	
*x*_3_^2^	0.0234	1	0.0234	9.6	0.0113	
Residual	0.0244	10	0.0024			
Lack of Fit	0.0196	5	0.0039	4.1	0.0739	
Pure Error	0.0048	5	0.001			

**Table 11:** Model reliability analysis.

Model	Std. Dev. /(MPa)	Mean	C.V. /(%)	R^2^	Adjusted R^2^	Predicted R^2^	Adeq Precision
Y_7d_	0.0494	1.51 MPa	3.27	0.9683	0.9398	0.7975	19.5081

**Table 12:** Prediction and verification test results under optimal conditions.

Factors			7d UCS		Absolute Relative Deviations/(%)
Moisture Content/(%)	Curing Agent Content/(%)	Mix-Grinding Time/(min)	Predicted	Actual	
18.51	11.46	26.48	1.74	1.72	1.15

**Table 13:** Qualitative and quantitative results of AMIW-stabilized soil by Rietveld method.

Minerals		Phase Contents/(wt.%)	
		7d	28d
		*R*_wp_ = 11.83% *R*_exp_ = 4.99%	*R*_wp_ = 12.14% *R*_exp_ = 5.16%
C-S-H	CaO·SiO_2_·*n*H_2_O	3.31 (6)	5.76 (5)
ettringite	3CaO·Al_2_O_3_·3CaSO_4_·32H_2_O	1.41 (2)	3.54 (2)
Quartz	SiO_2_	22.53 (3)	22.63 (3)
calcite	CaCO_3_	10.06 (2)	10.68 (2)
Chlorite	(Fe, Mg)_5_Al(Si_3_Al)O_10_(OH)_8_	18.55 (5)	18.14 (4)
Muscovite	KAl_2_(AlSi_3_O_10_)(OH)_2_	12.95 (3)	13.8 (3)
Plagioclase	(Na,Ca)(Si,Al)_4_O_8_	19.45 (7)	14.24 (4)
Microcline	KAlSi_3_O_8_	6.7 (3)	7.26 (3)
Anorthite	CaAl_2_Si_2_O_8_	5.03 (3)	3.96 (3)
Sum		99.98	100.01

**Table 14:** Pore structure of AMIW-stabilized soil.

Sample	Total Intrusion Volume (mg/L)	Porosity (%)	Bulk Density (g/mL)	Apparent Density (g/mL)	Average Pore Diameter (nm)
7 d	0.21	33.07	1.61	2.35	73.36
28 d	0.19	30.33	1.64	2.36	42.16
60 d	0.18	29.58	1.66	2.41	37.36

## Data Availability

The raw data supporting the conclusions of this article will be made available by the authors on request.
